# Cooperation of BMP and IHH signaling in interdigital cell fate determination

**DOI:** 10.1371/journal.pone.0197535

**Published:** 2018-05-17

**Authors:** Arunima Murgai, Sara Altmeyer, Stephanie Wiegand, Przemko Tylzanowski, Sigmar Stricker

**Affiliations:** 1 Institute for Chemistry and Biochemistry, Freie Universität Berlin, Berlin, Germany; 2 Development and Disease Group, Max Planck Institute for Molecular Genetics, Berlin, Germany; 3 Berlin-Brandenburg School for Regenerative Therapies, Charité Universitätsmedizin Berlin, Campus Virchow-Klinikum, Berlin, Germany; 4 Laboratory for Developmental and Stem Cell Biology, Department of Development and Regeneration, Skeletal Biology and Engineering Research Centre, University of Leuven, Leuven, Belgium; 5 Department of Biochemistry and Molecular Biology, Medical University of Lublin, Lublin, Poland; Laboratoire de Biologie du Développement de Villefranche-sur-Mer, FRANCE

## Abstract

The elaborate anatomy of hands and feet is shaped by coordinated formation of digits and regression of the interdigital mesenchyme (IM). A failure of this process causes persistence of interdigital webbing and consequently cutaneous syndactyly. Bone morphogenetic proteins (BMPs) are key inductive factors for interdigital cell death (ICD) in vivo. NOGGIN (NOG) is a major BMP antagonist that can interfere with BMP-induced ICD when applied exogenously, but its in vivo role in this process is unknown. We investigated the physiological role of NOG in ICD and found that *Noggin* null mice display cutaneous syndactyly and impaired interdigital mesenchyme specification. Failure of webbing regression was caused by lack of cell cycle exit and interdigital apoptosis. Unexpectedly, *Noggin* null mutants also exhibit increased *Indian hedgehog* (*Ihh*) expression within cartilage condensations that leads to aberrant extension of IHH downstream signaling into the interdigital mesenchyme. A converse phenotype with increased apoptosis and reduced cell proliferation was found in the interdigital mesenchyme of *Ihh* mutant embryos. Our data point towards a novel role for NOG in balancing *Ihh* expression in the digits impinging on digit-interdigit cross talk. This suggests a so far unrecognized physiological role for IHH in interdigital webbing biology.

## Introduction

Programmed cell death is a biological process essential for progressive sculpting and structuring of the developing autopod. Controlled cell death is evident in marked areas of the limb mesenchyme, primarily the anterior apoptotic zone (AAZ), posterior apoptotic zone (PAZ) and the interdigital apoptotic zone (IAZ). During the development of digits, the undifferentiated interdigital mesenchyme (IM) undergoes cell cycle withdrawal, senescence and apoptosis, which is crucial for individualization of digits and differential digit elongation [1–4]. Concomitantly, the initial digit condensations form and elongate by local recruitment of mesenchymal progenitors to a cartilage fate through elevated SMAD-dependent BMP signaling in antagonism with Wnt/β-catenin signaling [5–9]. In mice, controlled regression of the interdigital mesenchyme begins below the apical ectodermal ridge (AER) at embryonic day 12.5 (E12.5) and expands proximally by E14.5 [1,10]. Misregulation of interdigital cell death (ICD) typically results in failure of digit separation and consequently cutaneous syndactyly. Previous studies in mice and chicken have shown that interdigital cell fate is mainly governed by the interactions between bone morphogenetic protein (BMP), fibroblast growth factor (FGF) and retinoic acid (RA) pathways (Hernández-Martínez & Covarrubias, 2011).

BMPs have been shown to play a crucial role in regulating ICD in mice and chicken. *Bmp2*, *4* and *7* are expressed in the IM just preceding or during ICD [1,11–14]. Application of BMPs to this mesenchyme in chicken embryos results in accelerated cell death [13–16]. Likewise, the application of the BMP antagonist NOGGIN (NOG) prevents ICD in chicken limbs thus inducing syndactyly [17–19]. On the contrary, in mice, administration of NOG or the BMP-inhibitor dorsomorphin does not alter ICD [2]. However, limb mesenchyme-specific inactivation of *Bmp2/4* or interdigit-specific deletion of either *Bmpr1a*, *Bmp7* or *Bmp2/4* results in interdigital syndactyly in mice due to ICD reduction, indicating a direct role [20,21].

ICD is thought to be controlled by the interplay of signals originating from the interdigital mesenchyme and the AER. The AER is a specialized layer of ectodermal cells that controls limb outgrowth and patterning by expressing several key members of the FGF family [22,23]. FGFs, most importantly FGF8, promote cell survival in the nascent limbs [24]. Implantation of FGF8 beads in interdigital mesenchyme dramatically reduces ICD [2,16]. BMP and FGF signaling are involved in an antagonistic interplay during interdigit regression. For example, conditional inactivation of *Bmpr1a* in the AER, or ectopic expression of *Noggin* in the ectoderm, or ablation of the BMP target genes *Msx1/2*, all result in persistent *Fgf* expression [11,25,26]. Furthermore, downregulation of *Fgf* expression in the AER overlying the interdigit areas coincides precisely with the time of ICD induction [1]. Interestingly, prevalent Fgf8 expression in bats in concert with BMP inhibition mediated by the antagonist Gremlin leads to interdigital webbing [27]. Altogether, this suggests that BMPs induce ICD both directly and via an AER-*Fgf* regulatory loop.[21].

In addition, RA produced in the interdigital region by RALDH2 (encoded by *Aldh1a2*) is a potent inducer of cell death within the mesenchyme and can antagonistically regulate AER-Fgf8 expression, possibly via BMP induction [2]. The ectopic application of RA to interdigital regions in chicks induces interdigital tissue elimination which is preceded by upregulated expression of *Bmps* indicating that RA acts directly upstream of BMP signaling [28]. Similarly, the inactivation of RA signaling receptors *Rar* and *Rxr*, or of *Adlh1a2*, results in perturbed ICD and furthermore syndactyly [10,29,30]. Overall, the fine balance between BMP, FGF and RA signals determines the fate of the IM cells indicating that deregulating one or more of these signaling centers can result in syndactyly.

BMPs induce expression of their own antagonist NOG in the developing digits and the surrounding perichondrium, where NOG actively binds to BMPs in turn suppressing BMP signaling [31]. Inactivation of *Noggin* results in enhanced chondrogenesis resulting in enlarged cartilaginous condensations due to lack of BMP feedback inhibition [32].

In this study, we investigate the consequence of *Noggin* inactivation on interdigital regression in mice. Remarkably, Noggin deficient embryos show hallmarks of cutaneous syndactyly with failure in interdigital cell cycle withdrawal and apoptosis. No significant difference in interdigital pSMAD1/5/8 activity was detected indicating that canonical BMP/SMAD signaling is not directly responsible for this syndactyly phenotype. We observed that Noggin null mutants show leakiness of IHH signaling originating from the digits into the interdigital regions. In line with this, *Ihh* mutant embryos showed a contrasting phenotype with reduced interdigit proliferation and enhanced apoptosis. We propose that IHH is a novel member of the BMP, FGF, RA signaling loop participating in the specification and sculpting of the interdigital mesenchyme.

## Materials and methods

### Mice

This study was approved by the institutional animal welfare board of the Max Planck Institute for Molecular Genetics and the Landesamt für Gesundheit und Soziales Berlin (LAGeSo). Mouse lines used in this study have been described before: *Noggin* [32]; *Ihh* [33][34]. Mice were maintained in an enclosed, pathogen-free facility; mice were sacrificed by cervical dislocation. Experiments were performed in accordance with European Union regulations with permission from the Landesamt für Gesundheit und Soziales (LAGeSo) Berlin under licenses ZH120 and G0346/13. For each of the experiments, wildtype embryos were used as controls and compared against the desired mutant embryos (*Nog*^-/-^ or *Ihh*^-/-^).

### X-gal staining

Embryos were washed in PBS and fixed in fixing solution (0.2% glutaraldehyde, 2mM MgCl2, 5mM EDTA) at 4°C for 30min, followed by 3x5 min wash in wash buffer (2mM MgCl2, 0.01% sodium deoxycholate, 0.02% NonidentP40 in PBS). The embryos were stained in staining solution (1mg/ml X-Gal, 5mM K3Fe(CN)6, 5mM K4Fe(CN)6, 2mM MgCl2, in PBS) at 37°C shielded from light until the desired staining was obtained. The stained embryos were fixed for 30min in 2% PFA / 0.2%Glutaraldehyde fixed in PBS and imaged using a binocular microscope (Leica MZ 12).

### Whole-mount in-situ hybridization

For whole-mount in-situ hybridization, *Noggin* and *Ihh* mouse embryos of the stages E12.5-E14.5 were collected in PBS/DEPC and fixed overnight in 4% PFA/PBS. The embryos were washed in PBS (Tween 0.1%) for 2x15min and dehydrated using a methanol series: 2x15min 50% methanol and 100% methanol for 15min. The embryos were stored at -20°C until use. For hybridization, the embryos were incubated in a descending methanol series, washed twice with PBST and bleached in 6% Hydrogen peroxide in PBST for 1h at 4°C. The embryos were treated with proteinase K for the following durations-E12.5 for 5min, E13.5 for 8min and E14.5 for 12min. After thorough washing with PBST, PBST/glycine (2 mg / ml) and RIPA buffer, the embryos were fixed for 20min in 4%PFA/0.2% glutaraldehyde and washed several times in PBST. After prehybridization at 65°C in hybridization buffer (1ml of 1M Tris (pH 7.5), 12ml of 5M NaCl, 200μl of 0.5M EDTA, 1.25ml of 20% SDS, 25ml of 40% Dextran sulfate, 2ml of Denhardt's reagent, 2 ml of tRNA, 50 ml of formamide in 100 ml with DEPC-water) for at least 3 h, the embryos were incubated overnight with the desired probe at 65°C. For hybridization, the probes were diluted in hybridization buffer at a final concentration of 0.25μg/ml, denatured at 80°C for 5min, and then added the embryos. The unbound probes were removed the following day after washing with fresh hybridization buffer for 2x30min at 65°C. After the embryos had cooled to RT, RNaseA digestion was performed 37°C. For this the mixture was washed several times with formamide buffer at 65°C, initially with 1: 1 diluted with RNase wash buffer (5ml NaCl (5M), 500μl Tris (pH 7.5, 1M), 500μl 10% Tween20, make up to 50ml with H2O) and later diluted with (1: 1) MABT (100 ml of maleic acid (1M, pH 7.5), 30 ml of NaCl (5M), 10 ml of 10% Tween20, to 200 ml with H2O) followed by two wash steps with MABT. For saturation of nonspecific RNAs, the preparations were incubated for 1 h in 10% Boehringer Blocking Reagent in MABT and then incubated overnight at 4°C with anti-DIG-Fab antibody (1:5000) in 1% BBR / MABT on a shaker. Unbound antibody was removed the following day by washing with PBST/tetramisole (500mg/1l) 8x30min on a shaker at RT. To detect the antibody signal, embryos were washed 3× 20min in ALP buffer and then stained with BM Purple. The embryos were incubated at RT shielded from light until the desired staining was obtained. To preserve the signals, the embryos were washed 3×10min with ALP buffer and fixed in 4%PFA/PBS/0.2% glutaraldehyde. The embryos were imaged using the binocular microscope (Leica MZ 12).

### Tissue preparation

To prepare the tissue for the paraffin bedding, the limbs of 13.5 embryos were dissected and incubated overnight in 4%PFA/PBS at 4°C. On the following day the preparations were washed 2x10min in PBS, then incubated at RT for 1h in 50% EtOH, and subsequently dehydrated for 1h in 70% EtOH. The other necessary steps as were carried out with the help of the a paraffin-embedding work station, according to the following program: 3h 90% EtOH, 3h 95% EtOH, 2h with vacuum 100% EtOH, 2h with vacuum 100% EtOH, 2h with vacuum 100% EtOH, 15 min with vacuum utraclear (UC), 15min with vacuum UC, 30min with Vacuum UC, 3h with vacuum UC/paraffin, 3h with vacuum paraffin. The tissue was orientated as desired and embedded in liquid paraffin. The embedded limbs were cut into 6μm thick sections using a microtome (Mikrom HM 355 S or Reichert-Jung 2050 Supercut) and dried overnight at 37°C on a heating plate.

### BrdU incorporation and cell proliferation analysis

To determine the proliferation rate of cells in the interdigital mesenchyme, 5-bromo-2'-deoxy-uridine (BrdU, Roche) was intra-peritoneally injected into the pregnant females (50 mg kg^-1^) at the desired embryonic stage and the embryos were collected 1h later. After paraffin embedding, immunohistological analysis was performed on the tissue sections. The labeled transcript in proliferating cells could be detected with anti-BrdU antibody (described below). The proliferation rate was determined by counting the positive cells versus DAPI positive cells.

### Immunolabeling

The tissue sections were deparaffinized and rehydrated using the following steps: 45 min in UC, min in 100% EtOH, 2min in UC, 2min in 100%EtOH, 2min in UltraClear, 5min in 100% EtOH, 5min in 90% EtOH, 5min in 70% EtOH, 15min in H2O bidest. The slides were then placed in DAKO buffer, pH9 and heated in the microwave for 3min twice. The samples were then allowed to stand in the hot buffer for 30min at RT cool down. The tissue was then permeablized in 0.2% of TritonX in PBS for 15min RT and blocked with 5% goat serum/0.2% Tween in PBS for 1h at RT. Limb sections were stained for the following in blocking solution overnight at 4°C—(rabbit-anti-)phospho-Smad1/5/8 (CST-9511L, 1:200), (mouse-anti-)Sox9 (abcam ab76997, 1:150), (rabbit-anti-)Caspase3 (CST-96645, 1:200) and (mouse-anti-)Brdu (Roche-11170376001, 1:50). This was followed by incubation with the secondary antibody in blocking solution for 30 min at RT. For phosphor-Smad1/5/8 an additional tyramide signal amplification step was performed using Tyramide Signal Amplification kit (Perkin Elmer) as per the manufacturer’s instructions. The slides were covered in Fluoromount G and imaged under the fluorescence microscope (Zeiss Axiovert 200).

### Real-time PCR

Whole hand plates were dissected at E12.5 or E13.5 avoiding the wrist region. Microdissection of digits or interdigit mesenchyme from E12.5 or E13.5 embryos was performed with tungsten needles. For digit / interdigit analysis tissue obtained from all individual digits / interdigits from one handplate was pooled and treated as one biological sample. Total RNA extraction was performed using RNeasy micro kits (Qiagen) according to manufacturer’s instructions; 1μg of RNA was subjected to reverse transcription using High Capacity cDNA Reverse Transcription Kit (Applied Biosystems). Gene expression was assessed using Taqman Gene Expression Assays (Applied Biosystems) on a 7900HT Real Time PCR system (Applied Biosystems). Data were acquired and analyzed using SDS 2.0 software (Applied Biosystems). Transcript expression levels were calculated as mean normalized expression (MNE) ratios referred to GAPDH as housekeeping gene using the ΔΔCT method. Analysis was performed on three independent biological samples obtained from different embryos. Primer sequences and gene accessions numbers are depicted in Table 1.

**Table 1:** Primer sequences for real-time RT-qPCR.

Target	Sequence 5’– 3’	Accession number
*Ihh*	**F** GCCGACCGCCTCATGAC **R** CATGACAGAGATGGCCAGTGA	NM_001166361.1
*Gli1*	**F** CCCATAGGGTCTCGGGGTCTCAAAC **R** GGAGGACCTGCGGCTGACTGTGTAA	NM_001313683.1
*Ptch1*	**F** TGCTGTGCCTGTGGTCATCCTGATT **R** CAGAGCGAGCATAGCCCTGTGGTTC	NM_010296.2
*Bmp2*	**F** GTACCGCAGGCACTCAGG **R** AAGTTCCTCCACGGCTTCTT	NM_001328514.1
*Bmp4*	**F** CCGGATTACATGAGGGATCT **R** CCAGATGTTCTTCGTGATGG	NM_001316360.1
*Bmp7*	**F** CTACATGAACGCCACCAACC **R** AGGACAGAGATGGCGTTGAG	NM_007557.3
*Grem1*	**F** CAAGGCTCAGCACAATGACT **R** GACTCAAGCACCTCCTCTCC	NM_011824.4
*Gapdh*	**F** GGGAAGCCCATCACCATCTT **R** CGGCCTCACCCCATTTG	NM_008084
*Chdl1*	**F** TCCAAGTGCCAGGAGTAACC **R** AACTCGTCCATGCTTGTGC	NM_204171
*Chdl2*	**F** CAAGAAGCGCAGAACTACAGG **R** TGTCATCCTCACCTCTGACG	NM_417245
*Tsg*	**F** CGTTGCAGAAGAGCTGTCG **R** GAGACGTTCTGATGCTGTGG	NM_204198

## Results

### Digit formation and cutaneous syndactyly in *Noggin* null embryos

**Fig 1:**
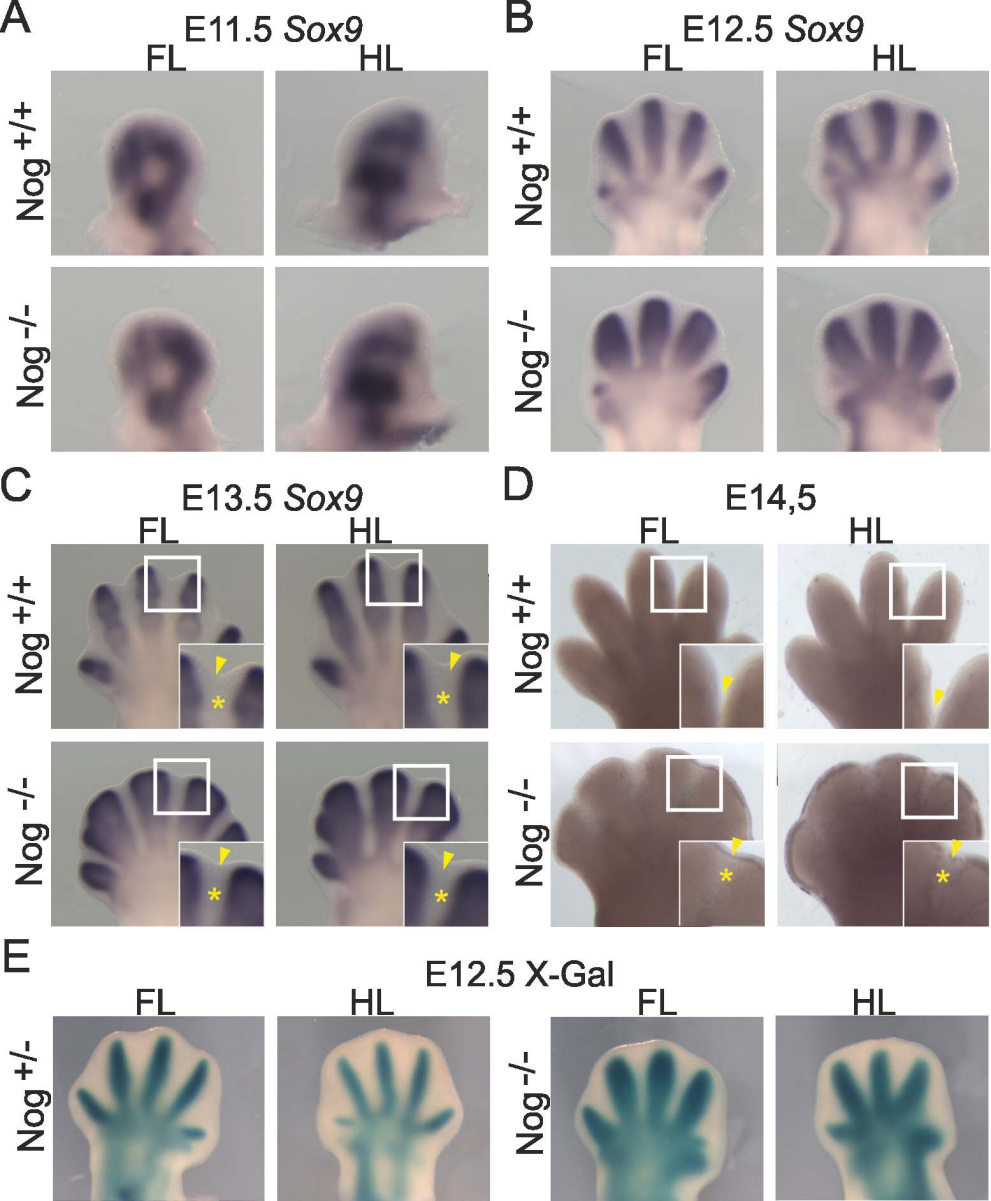
Digit and interdigit formation in *Nog*^*-/-*^ embryos. Cartilage condensation was assessed by whole-mount in-situ hybridization for *Sox9* (A-C) and X-Gal staining detecting β-Galactosidase expressed from the *Noggin* locus in *Nog*^*+/-*^ or *Nog*^*-/-*^ embryos (E). (D) Shows unstained autopods. Stages are indicated; FL: forelimb, HL: hindlimb. Indentation between digits is marked by an arrowhead, the interdigital mesenchyme is marked by an asterisk in (C and D).

Digit formation in amniotes begins with the condensation of chondrocytes forming the digit rays followed by endochondral ossification. Subsequent segmentation generates digital synovial joints. Nascent chondrogenic condensations of the digits appear around E11.5 marked by expression of *Sox9*. *Noggin* null mice displayed a normal *Sox9* condensation pattern at E11.5 (Fig 1A). At E12.5, where individual digit condensations are visible, marked lateral expansion of the condensations especially in the proximal parts of the condensations was seen in *Nog*^-/-^ mutants, which became more pronounced by E13.5 (Fig 1B and 1C). However, a stripe of Sox9 negative tissue was detected between the condensations in *Nog*^-/-^ embryos (Fig 1C, asterisks). During this time, the regression of the interdigital mesenchyme begins, as visible by distal indentation between the digit condensations [1], a process that was less prominent in the Noggin null mice (Fig 1C, arrowheads), and cutaneous syndactyly became clearly visible by E14.5 (Fig 1D).

Since *Noggin* itself is widely used as a cartilage differentiation marker [32,35], we used the LacZ reporter allele inserted into the *Noggin* locus to independently trace digit condensations [32,35]. In agreement with previous data [32] we found that *Noggin* expression, just like *Sox9*, was restricted to the digit condensations and not found in IM (Fig 1E). It is to be noted that in the control embryos (*Nog*^*+/LacZ*^), β-Galactosidase is expressed from one allele, and from two alleles in the mutant embryos (*Nog*^*LacZ/LacZ*^). In summary, despite the expanded condensations, the interdigital mesenchyme of *Nog*^*-/-*^ embryos remains devoid of ectopic chondrogenic cell differentiation.

### *Noggin* null embryos display impaired interdigit marker expression

ICD is regulated by complex molecular interactions involving three main signaling pathways- BMPs, RA and FGFs. To analyze this process in the *Nog*^*-/-*^ mutants we investigated expression of interdigital markers connected to these pathways. *Msx* genes encode homeodomain transcription factors and are downstream effectors of the BMP signaling pathway. Analysis of *Msx1*^*-/-*^*;Msx2*^*-/-*^ double knockout mice revealed that *Msx1/2* act downstream of BMP4 in the IM and play a role in AER maturation and regression [26]. *Msx* genes can be induced and maintained mainly by BMPs, but also by FGFs [36].

**Fig 2:**
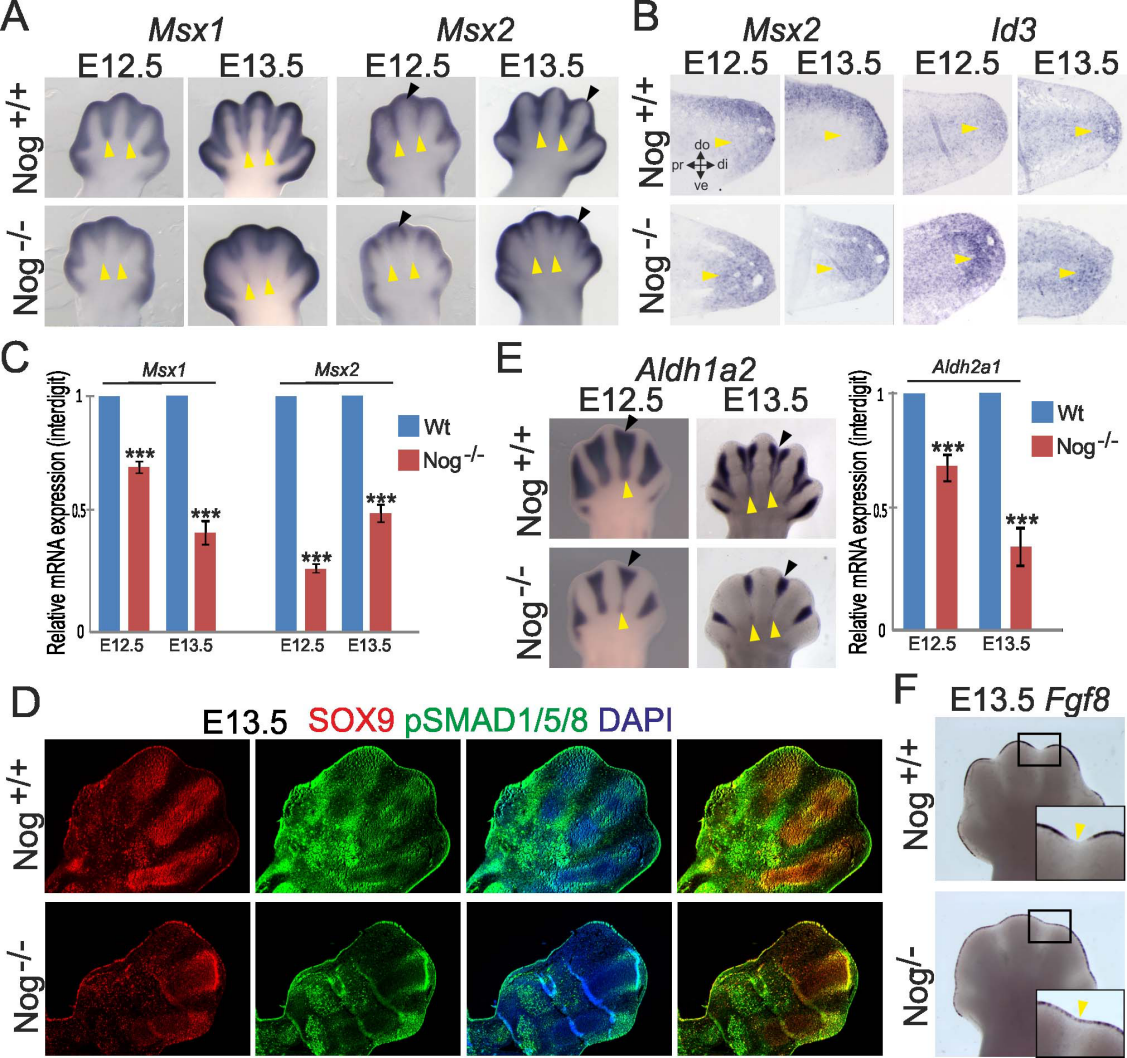
Noggin mutants display impaired interdigit marker expression. (A) Whole-mount ISH for *Msx1* and *Msx2* shows impaired expression in proximal interdigital mesenchyme (yellow arrowheads) for both genes; *Msx2* appears increased at the digit tips in *Nog*^*-/-*^ hand plates (black arrowheads). (B) Section ISH for *Msx2* and *Id3* on longitudinal hand plate sections, digit 3 is shown; orientation as indicated di: distal, do: dorsal, pr: proximal, ve: ventral). *Msx2* and *Id3* show increased expression at the digit tips (arrowheads). (C) Expression of *Msx1* and *Msx2* was analyzed by real-time qPCR on mRNA extracted from microdissected interdigit tissue. (D) BMP canonical signaling was assessed by immunolabeling for phosphorylated SMADs (pSMAD1/5/8). No aberrant pSMAD signal is visible in the interdigital region in *Nog*^*-/-*^ hand plates. Increased signaling is apparent at the circumference of cartilage condensations in *Nog*^*-/-*^ hand plates. (E) *Nog*^*-/-*^ hand plates exhibit impaired *Aldh1a2* expression, especially in the proximal interdigital mesenchyme (yellow arrowheads), at E13.5 also in the distal interdigital mesenchyme (black arrowheads). (F) Persistent expression of *Fgf8* in the AER overlying the interdigits (yellow arrowheads) in *Nog*^*-/-*^ hand plates.

Whole-mount in-situ hybridization showed that *Nog*^*-/-*^ mutants displayed a reduction in *Msx1* and *Msx2* expression in the proximal interdigital region. For *Msx2*, a reduction of expression in the distal interdigital region was also observed (Fig 2A), however the expression domain distal to the growing condensation appeared unaffected or even increased. We additionally performed ISH on tissue sections that confirmed elevated *Msx2* expression at the tip of the distal cartilaginous condensation. Another early BMP target gene, *Id3*, expressed in distal mesenchyme overlapping with the phalanx-forming region, also showed increased expression in *Nog*^*-/-*^ embryos (Fig 2B). Quantification of *Msx1* and *Msx2* expression levels via real time RT-qPCR on mRNA extracted from microdissected hand plate interdigit tissue confirmed an overall downregulation of both genes in the interdigit region (Fig 2C). Altogether, these results point to decreased BMP signaling in the proximal interdigits, but increased signaling at the digit tips encompassing the phalanx-forming region.

SMADs are downstream intracellular transducers of BMP signaling. Active pSMAD1/5/8 signaling is known to be present in the interdigital mesenchyme and in the developing cartilage anlage [37]. Within the Sox9-expressing cartilage elements of the *Nog*^*-/-*^ embryos, no aberrantly high pSMAD1/5/8 signaling was detected, with the exception of the condensation borders (Fig 2D). Here, active chondrogenesis is taking place which most likely accounts for the expansion of the digits. Strong pSMAD1/5/8 signal was detected within the phalanx-forming region at the distal tip of the enlarged condensation in *Nog*^-/-^ embryos (Fig 2D) concomitant with the increase in *Id3* expression. *Nog*^-/-^ embryos did not show any detectable ectopic pSMAD1/5/8 signal in the interdigits, neither proximal nor distal (Fig 2D). Thus, at 12.5 dpc, loss of *Noggin* did not increase canonical BMP/SMAD signaling in the interdigital region of the mouse autopod.

Retinoic acid is known to be a potent cell death inducer in IM as its application to the interdigit induces interdigital regression whereas a RA inhibitor impedes ICD [2,28]. We investigated the expression of *Aldh1a2*, which encodes RALDH2, the key enzyme responsible for RA synthesis [29]. *In situ* hybridization for *Aldh1a2* showed that its expression domain was reduced in the proximal interdigital mesenchyme of *Nog*^*-/-*^ embryos (Fig 2E), thus indicating reduced RA synthesis in line with reduced interdigital marker expression in this region. In the distal IM, however, *Aldh1a2* appeared to be expressed normally at E12.5, but was decreased at E13.5 (Fig 2E). *Aldh1a2* is regulated by HOXD13, and reduced *Aldh1a2* expression in *Hoxd13* mutant mice was linked to synpolydactyly [38]. However, *Hoxd13* showed a normal expression in *Nog*^-/-^ embryos (S1A Fig).

FGFs act as survival factors and antagonize RA during proximal-distal limb outgrowth and ICD. The onset of cell death in the mouse distal IM coincides with the regression of the AER and thus the loss of FGF8 signals [1]. Enhanced BMP signaling reduces *Fgf* expression in the AER [39,40]; unexpectedly we found that *Nog*^-/-^ embryos exhibited sustained *Fgf8* expression in the AER overlying the interdigit region (Fig 2F).

Overall, the deletion of *Noggin* affects RA and down-stream BMP signaling while concurrently *Fgf8* expression in the AER overlying the interdigits is maintained, opposite to the expectation of exacerbated BMP signaling after antagonist removal.

### Increased Indian hedgehog signaling in *Nog*^*-/-*^ embryos

**Fig 3:**
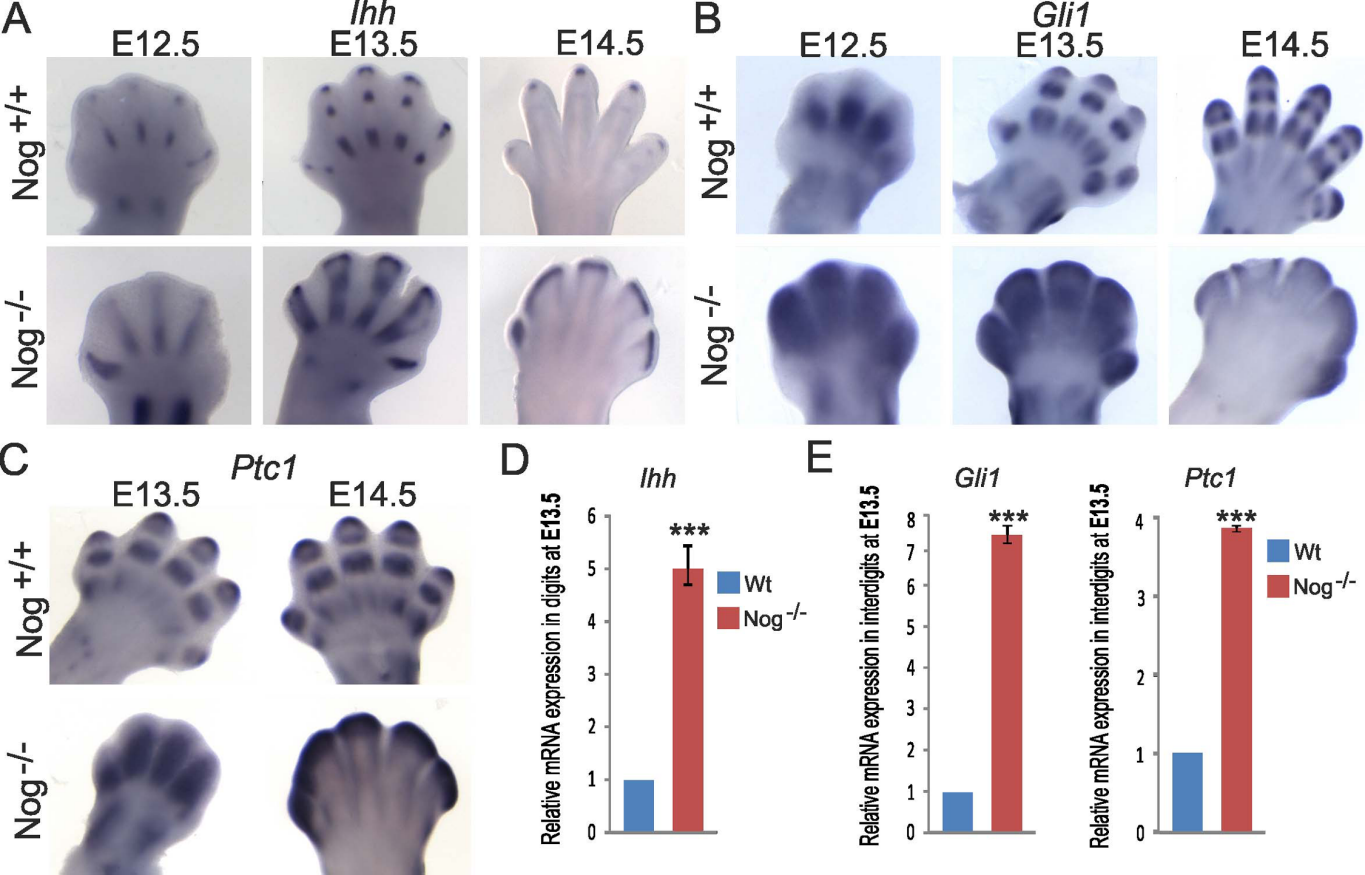
Increased *Indian hedgehog* (*Ihh*) expression and downstream signaling in *Nog*^*-/-*^ hand plates. The expression of *Ihh* (A), *Gli1* (B) and *Ptc1* (C) was assessed by Whole-mount ISH. Note the strong upregulation of *Ihh* expression in cartilage condensations and the diffuse expression of the IHH targets *Gli1* and *Ptc1* in interdigital mesenchyme in *Nog*^*-/-*^ autopodes. (D, E) E13.5 hand plates were microsurgically dissected into interdigit and digit mesenchyme. Quantitative RT-PCR confirms increased *Ihh* expression in digit condensations (D) and increased expression of *Ptc1* and *Gli1* in interdigit mesenchyme (E). Error bars represent S.E.M. T-test: * = p<0.05; ** = p<0.01; *** = p<0.001 (n = 3).

*Nog*^-/-^ embryos exhibit fused digit joints as a result of over-proliferation and faulty differentiation of chondrocytes [32], a feature also observed in mice overexpressing *Ihh* in the growth plate [41–43]. Indian Hedgehog (IHH) is expressed in pre-hypertrophic chondrocytes and it regulates proliferation and differentiation of chondrocytes [33,44]. *Nog*^-/-^ embryos showed a striking increase in *Ihh* expression in the digits at E12.5 and E13.5, whereby the *Ihh* expression domain extended along the digit condensation (Fig 3A) confirming previous reports [32,45]. Especially in the most distal condensation, the *Ihh* domain was laterally expanded in Nog^-/-^ embryos. IHH signaling from this region was involved in chondrogenic recruitment of mesenchymal progenitors to the cartilage condensation [46] and may in part explain the augmented cartilage formation in the digits. In wild type mice, the IHH downstream targets *Gli1* and *Ptc1* are expressed mainly in the digits and the perichondrium. In *Noggin* mutants the expression domains of both *Gli1* and *Ptc1* were broader and less sharply defined (Fig 3B and 3C), indicating an expanded IHH signaling range into the prospective interdigital mesenchyme. Increased *Ihh* expression in digit condensations of *Nog*^-/-^ embryos hand plates and well as expression of IHH downstream targets *Gli1* and *Ptc1* in the interdigit mesenchyme was confirmed by real time RT-qPCR on mRNA extracted from microdissected hand plate digit and interdigit tissues (Fig 3D and 3E). To define the interrelationship between NOGGIN/BMP and IHH signaling, interdigit regression was from here on investigated comparatively in both *Nog*^*-/-*^ and *Ihh*^*-/-*^ embryos.

### Apoptosis and proliferation in the interdigit mesenchyme

**Fig 4:**
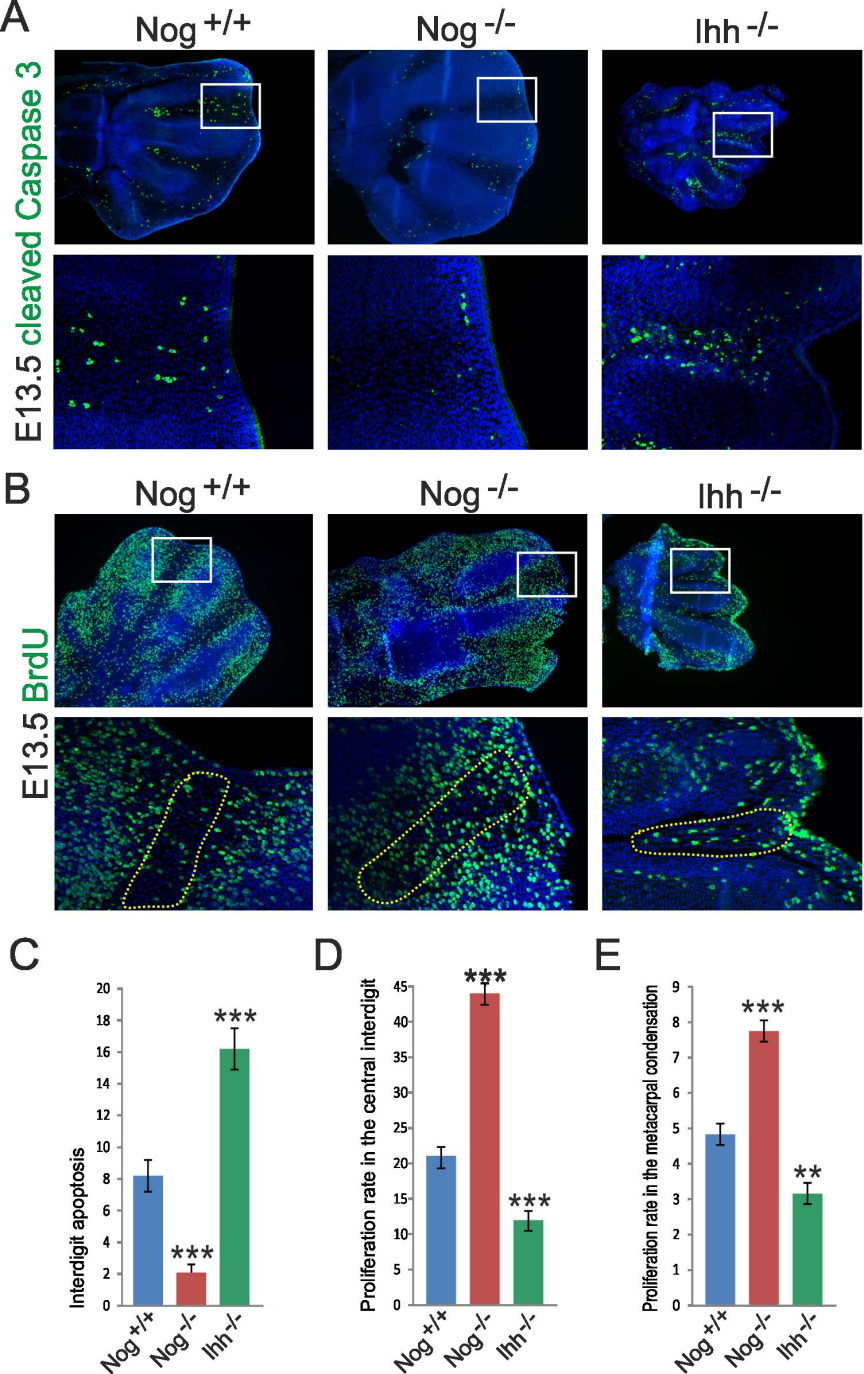
Apoptosis and proliferation of interdigital cells are altered conversely in *Nog* and *Ihh* mutants. (A) Apoptosis was assessed by immunolabeling for cleaved Caspase 3. Boxed areas showing the distal interdigital mesenchyme are shown as magnifications below. (B) Cell proliferation was assessed by immunolabeling for 5-Bromodesoxyuridine (BrdU) incorporated into the DNA of dividing cells. Boxed areas showing the distal interdigital mesenchyme are shown as magnifications below. (C) Quantification of interdigital apoptosis depicted as cleaved Caspase 3-positive cells / total number of interdigital cells. (D) Quantification of interdigital proliferation depicted as BrdU-positive cells / total cells. Quantification of proliferation was performed in the central interdigit (dashed line in (B)). (E) Quantification of proliferation within digit metacarpal condensations depicted as BrdU-positive cells / total cells. Error bars represent S.E.M. T-test: * = p<0.05; ** = p<0.01; *** = p<0.001 (n = 3).

Interdigit regression is a result of decreased proliferation and a concomitant surge in cell death of the undifferentiated interdigital mesenchymal region [47,48]. As opposed to the chick, apoptosis in the mouse is taking place mainly in the distal part of the interdigital mesenchyme [2]. We assessed apoptosis in the interdigital mesenchyme using immunolabeling against active Caspase 3, a key signal transducer of the canonical mitochondrial apoptotic pathway [49]. A remarkable decrease in cell death was apparent in the interdigital mesenchyme of *Nog*^-/-^ embryos in both proximal and distal regions (Fig 4A and 4C). Intriguingly, *Ihh*^*-/-*^ embryos showed an opposite phenotype with an increase in interdigital apoptosis (Fig 4A and 4C). Next, BrdU labeling was used to assess the proliferation rate of the interdigital mesenchyme. In wild type mice, reduced BrdU signal indicating cell cycle withdrawal was observed in the central interdigit (Fig 4B and 4D). *Nog*^-/-^ embryos showed a significant increase in interdigital cell proliferation, conversely, *Ihh*^*-/-*^ embryos showed reduced proliferation (Fig 4B and 4D). Concomitantly, *Nog*^-/-^ embryos exhibited increased proliferation within the chondrogenic condensation (Fig 4E) likely contributing the expansion of the chondrogenic anlagen. In *Ihh*^*-/-*^ embryos the proliferation rate in chondrogenic condensations was reduced (Fig 4C) as was shown before [33]. These results suggest that increased proliferation concomitant with reduced apoptosis of interdigital cells causes the failure of interdigit regression in *Nog*^-/-^ embryos.

Unexpectedly, IHH appears to be required for maintaining the interdigital mesenchyme in a proliferative state and preventing apoptosis thus indicating a physiological role for IHH in regulating interdigital fate.

### BMP expression in the interdigit region

**Fig 5:**
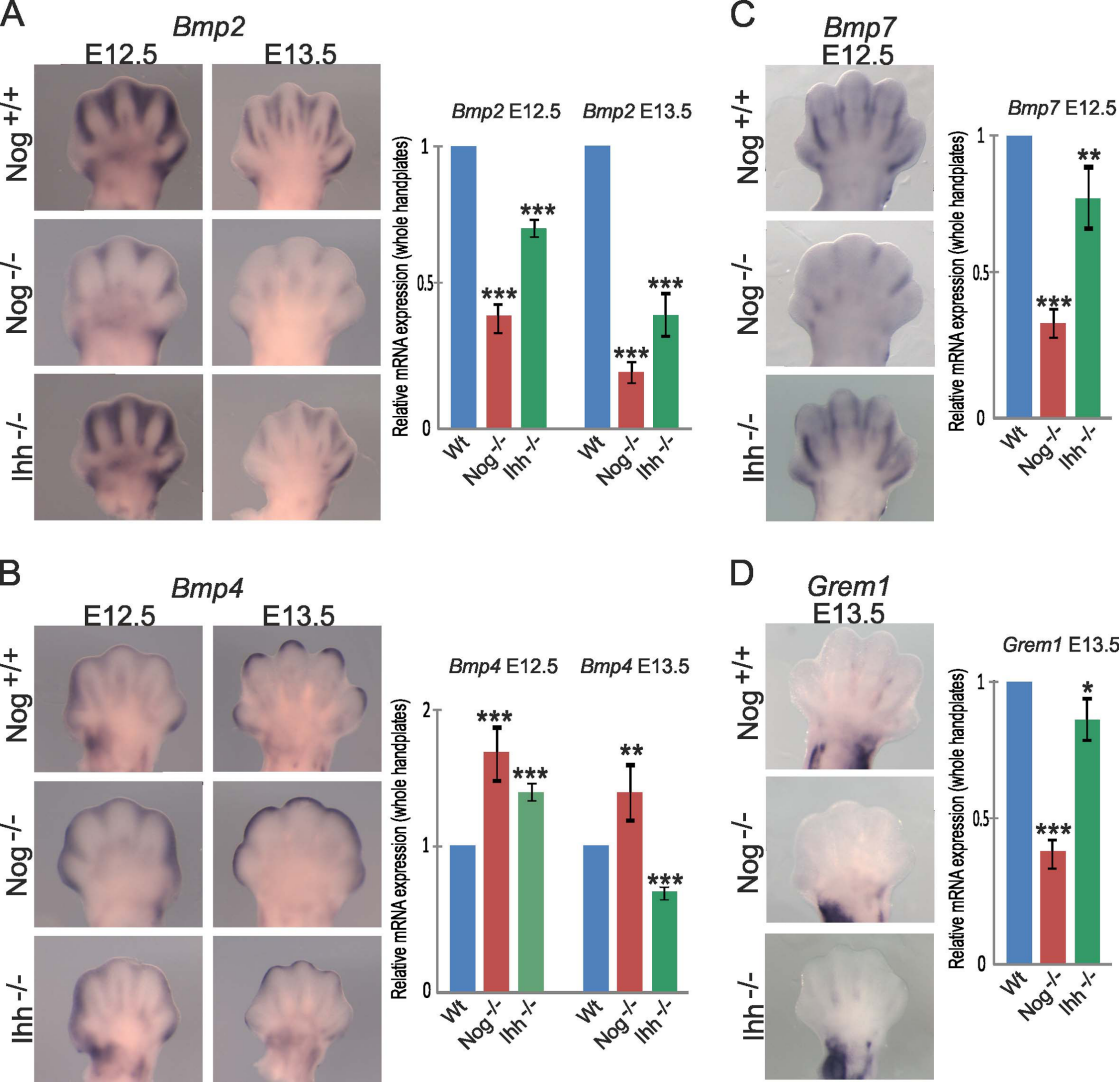
Expression of *Bmps* is affected in *Nog* and *Ihh* mutants. Expression of *Bmp2* (A), *Bmp4* (B), *Bmp7* (C) and the BMP antagonist *Grem1* (D) were as assessed by Whole-mount ISH and RT-qPCR on mRNA extracted from whole hand plates; stages as indicated. Error bars represent S.E.M. T-test: * = p<0.05; ** = p<0.01; *** = p<0.001 (n = 3).

Several members of the BMP family are known to play a role in ICD, among which BMP2, BMP4 and BMP7 are the most prominent regulators [20,21]. In mouse, the expression of *Bmp2* and *Bmp7* coincides with interdigital mesenchyme during ICD, while *Bmp4* is predominantly present in the mesenchyme underlying the distal ectoderm [1]. The expression of *Bmp* genes was altered in *Nog*^-/-^ embryos, whereby, *Bmp2* and *Bmp7* were downregulated, while *Bmp4* expression appeared slightly increased (Fig 5A–5C). High *Bmp4* expression as seen on the digit tips of the *Nog*^-/-^ embryos might be involved in sustaining digit growth and promoting mesenchymal cartilage differentiation at the distal tips of the digits [1]. It was suggested early on that the expansion of cartilage anlagen occurs as a result of increased recruitment of progenitors and changes in the rates of proliferation [32]. This notion is supported by the enhanced expression of BMP targets *Msx2* and *Id3* as well as the increased pSMAD1/5/8 signal specifically in this region of the *Nog*^-/-^ embryos (Fig 2B and 2D).

It is possible that the reduction in interdigital *Bmp* expression, particularly of *Bmp2* and *Bmp7* in the interdigit, contributes to reduced ICD. However, in IHH mutants, the expression of *Bmp2* and *Bmp4* also was downregulated at E13.5 while *Bmp7* appeared relatively unchanged (Fig 5A–5C). Among the BMP antagonists, Gremlin 1 is known to be expressed in the interdigit during ICD exerting an anti-apoptotic effect. The expression of *Grem1* is restricted to the proximal interdigit mesenchyme and may explain the reduced proximal ICD in mice [11] and webbing in ducks [17]. In both *Nog*^*-/-*^ and *Ihh*^*-/-*^ embryos *Grem1* did not show a compensatory upregulation (Fig 5D) nor did other known BMP antagonists like *Chdl1*, *Chdl2* and *Tsg* excluding them as the cause for syndactyly (S1B Fig).

## Discussion

In this study, we provide evidence for an interaction between the BMP and IHH signaling pathways in regulating interdigital cell fate. We show that *Noggin* null embryos initially form interdigital anlagen, but later display early hallmarks of cutaneous syndactyly with impaired interdigital marker expression, maintenance of cell proliferation and lack of apoptosis induction. The scope of this study is limited to prenatal development since *Noggin* mutant embryos are postnatally lethal.

In humans, loss of function mutations in *NOG* are well known to cause two distinct autosomal dominant developmental disorders, proximal symphalangism (SYM1A, MIM #185800) and multiple synostosis syndrome (SYNS1, MIM #186500) [50,51]. In addition, dominant *NOG* mutations cause Brachydactyly type B2 (BDB2, MIM #611377). Of note, apart from brachydactyly and distal symphalangism, BDB2 patients frequently exhibit cutaneous syndactyly of either or both hands and feet [52], altogether indicating a physiological role for NOG in digit / interdigit coordination.

Interdigital cell death is regulated by a balance between interdigital BMPs and RA signaling, which promote apoptosis, and AER-derived FGF8 supporting survival of the undifferentiated mesenchymal cells. Retinoic acid affects apoptosis either directly or by FGF and BMP signals [21]. RA can act upstream of BMP signaling by inducing interdigital BMPs, while simultaneously repressing the chondrogenic potential of BMPs by upregulating *Msx* genes thereby altogether supporting ICD [28]. It is known that mice lacking RA synthesizing enzymes or RA receptors display syndactyly and lack the expression of *Bmp7* [29,30,53], vice-versa RA can induce the expression of *Bmp7* in mice [2]. Moreover, conditional inactivation of *Bmp7* in the interdigital mesenchyme has shown that it is the only known *Bmp* gene strictly required for interdigital apoptosis [21]. *Noggin* null mice show a reduction of the *Aldh1a2* expression domain as well as *Bmp7* expression especially in the proximal interdigital mesenchyme. This in consequence likely leads to downregulation of *Msx* genes in this region resulting in aberrant chondrogenic commitment of mesenchymal cells as well reduced ICD. Notably, *Noggin* deficiency did not lead to apparent deregulation of pSMAD signaling in the interdigital mesenchyme indicating the involvement of alternative BMP downstream pathways in regulating interdigital apoptosis as suggested before [21].

In *Nog*^-/-^ embryos the AER overlying the interdigit regions failed to regress at E13.5 hence providing continuous FGF8 ‘survival signal’ to the underlying mesenchyme. *Fgf* expression in the AER is negatively regulated by BMP signaling [11]. *Nog*^-/-^ mutants showed reduced expression of *Bmp2* and *Bmp7* in the interdigital mesenchyme. However, mouse mutants with reduced Bmp expression in the interdigit mesenchyme did not exhibit maintenance of *Fgf8* expression in the overlying AER [21]. Furthermore, genetic evidence indicates that AER-expressed BMPs may be mainly responsible for balancing *Fgf8* expression [54,55]. This indicates that reduced mesenchymal BMP expression may not be the underlying cause of AER-*Fgf* maintenance in *Noggin* mutants.

Within the digit condensations, the loss of NOG led to expanded *Ihh* expression domains. *Ihh* is known as a downstream target of BMP signaling in cartilage [16,56], thus this likely is a direct effect of increased BMP signaling in the condensations. IHH was involved in positive regulation of chondrogenic commitment of mesenchymal progenitors to the digit condensations. Mutations in human IHH underlie brachydactyly type A1 (BDA1, MIM #112500) [57], caused by reduced chondrogenic recruitment into the digit anlage by BMP signaling in the PFR [7,46]. In addition, IHH is known to regulate chondrocyte proliferation and differentiation [33,44] in concert with BMP signaling [42]. Thus, it appears likely that the increased IHH signaling in *Nog*^-/-^ embryos contributes to the increase in cartilage condensation size by both fostering chondrogenic recruitment of mesenchymal progenitors as well as via induction of chondrocyte proliferation.

Importantly, the signaling activity of IHH (indicated by the expression of downstream targets *Ptc1* and *Gli1*) extended beyond the digit margins in *Nog*^-/-^ embryos. Duplications at the human *IHH* locus cause syndactyly in addition to craniofacial malformations [58,59]. Using mouse models recapitulating the human duplications, it was shown recently that this results in a reshuffling of the *Ihh* regulatory landscape leading to overexpression of *Ihh* specifically in the distal phalanges concomitant with an expansion of the distal IHH signaling range and decreased ICD [60]. This is in line with our hypothesis of an aberrant cross-tissue signaling between digits and interdigits in *Noggin* mutants involving IHH originating from the digit condensations. This suggests that deletion of *Ihh* in *Noggin* mutants might result in at least partial rescue of the syndactyly phenotype. We analyzed *Nog*^*-/-*^*;Ihh*^*+/-*^ mutants and observed no significant difference in the expression of *Msx1*, *Msx2* and *Aldh1a2* (S2 Fig). Loss of a single *Ihh* allele may not cause sufficient reduction of *Ihh* expression. We were, however, not able to retrieve *Nog*^*-/-*^*;Ihh*^*-/-*^ mutants at E13.5, presumably due to early lethality.

The mechanism by which IHH signaling prevents interdigital cell cycle withdrawal and ICD remains unknown. In the chick, application of beads soaked in Sonic hedgehog (SHH) implanted into the interdigital mesenchyme led to maintenance of *Fgf8* expression in the AER by an unknown mechanism [23] pointing towards an indirect AER-mediated effect. However, mice with decreased ICD due to distal *Ihh* overexpression did not show aberrant *Fgf8* expression [60]. This strongly argues that IHH has a direct, AER-independent effect on the interdigital mesenchyme. In this regard, the sustained Fgf8 expression we observed in *Noggin* mutants may be dispensable for the lack of interdigit regression. It is known from several cellular contexts that hedgehog signaling promotes cell proliferation and survival [61] in line with a direct effect on interdigital cells. Notably we found that *Ihh* mutant mice exhibit increased interdigital cell death and decreased interdigital proliferation, supporting a physiological role of IHH signaling in the interdigital mesenchyme. A major role of NOG in cartilage condensation may thus be to prevent overshooting IHH expression. This would help prevent exacerbated cartilage condensation and proliferation and thereby allow for interdigit mesenchyme cell cycle exit and apoptosis.

## Conclusions

In summary, this study argues that syndactyly in *Noggin* null embryos is directly caused by an intrinsic defect in interdigital mesenchyme cell fate regarding cell cycle withdrawal and apoptosis induction, but is not secondary to ectopic chondrogenesis. Opposing effects on interdigital apoptosis and proliferation in *Nog* and *Ihh* mutants support the view that IHH signaling from the condensation may have a direct role in regulating interdigital cell fate and that the upregulation of IHH signaling in *Noggin* mutants is causative for the defect in interdigit regression.

## Supporting information

S1 Fig Unchanged expression of Hoxd13 and BMP antagonists in Noggin deficient embryos. (A) *Hoxd13* expression was analysed by whole-mount in-situ hybridisation on E13.5 wild type and *Nog*^*-/-*^ autopodes. FL: forelimb; HL: hindlimb. (B) The mRNA expression levels of the BMP antagonists *Chdl1*, *Chdl2* and *Tsg* were analysed by quantitative real-time PCR on mRNA extracted from whole wild type or Nog^-/-^ hand plates.
(TIF)

S2 Fig Analysis of *Nog* / *Ihh* compound mutants. *Nog*^*+/+*^*;Ihh*^*+/-*^ (control), *Nog*^*-/-*^*;Ihh*^*+/+*^ (normal *Nog* KO) and *Nog*^*-/-*^*;Ihh*^*+/-*^ (compound mutant: *Nog* KO lacking one allele of *Ihh*) were analysed via whole-mount in-situ hybridisation for the expression of interdigit markers *Msx1*, *Msx2* and *Aldh1a2*. No amelioration of the *Nog* KO phenotype can be seen in compound mutants. Proximal interdigital expression domains of *Msx1*, *Msx2* and *Aldh1a2* that are affected in Nog mutants are indicated by arrowheads.
(TIF)
