# Supplementary figures and images for: Cooperation of BMP and IHH signaling in interdigital cell fate determination
Source: PLoS One. 2018 May 17;13(5):e0197535. doi: 10.1371/journal.pone.0197535 (PMC5957397; doi:10.1371/journal.pone.0197535)

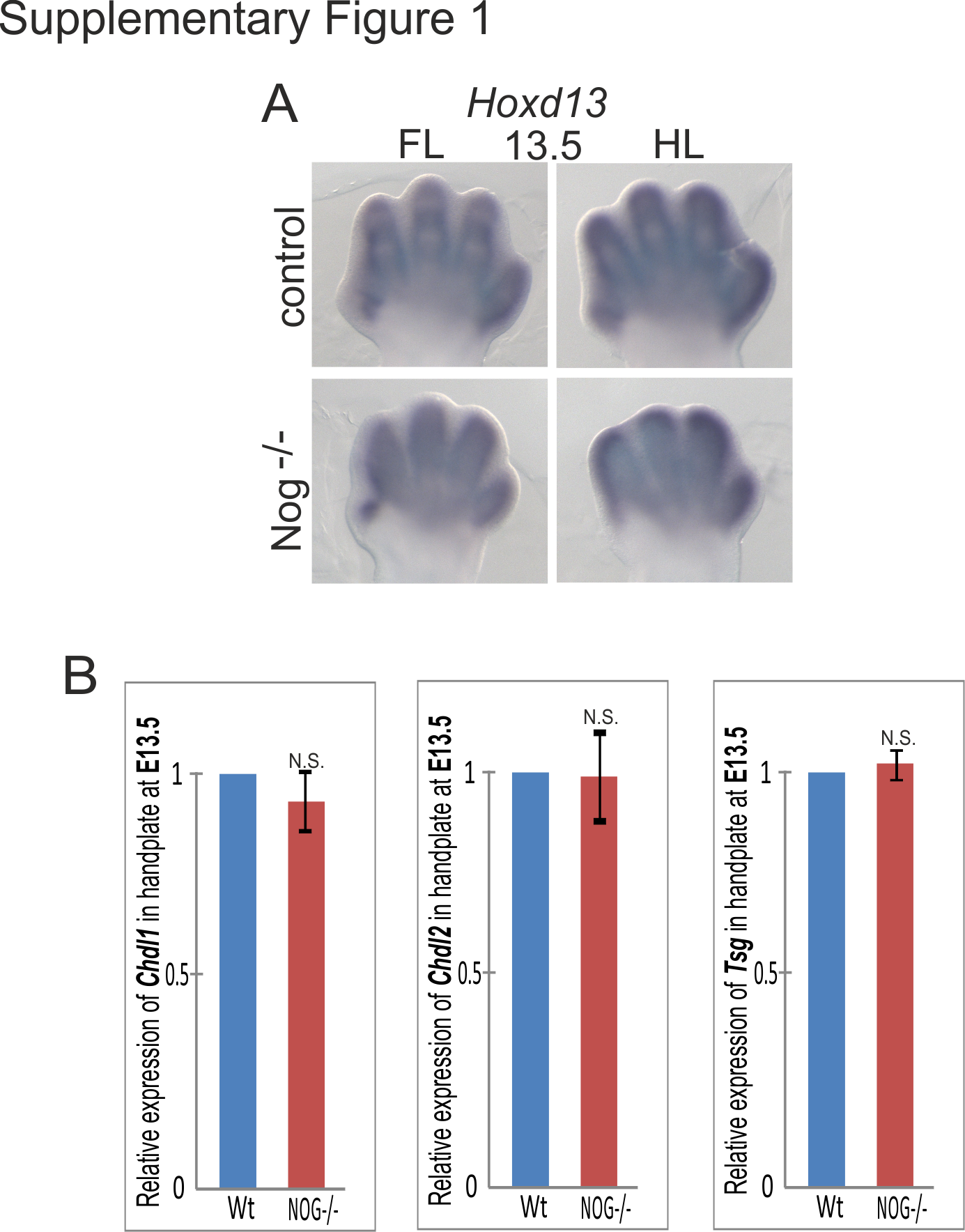

Supplement: S1 Fig — (A) Hoxd13 expression was analysed by whole-mount in-situ hybridisation on E13.5 wild type and Nog-/- autopodes. FL: forelimb; HL: hindlimb. (B) The mRNA expression levels of the BMP antagonists Chdl1, Chdl2 and Tsg were analysed by quantitative real-time PCR on mRNA extracted from whole wild type or Nog-/- hand plates. (TIF) [file pone.0197535.s001.tif]

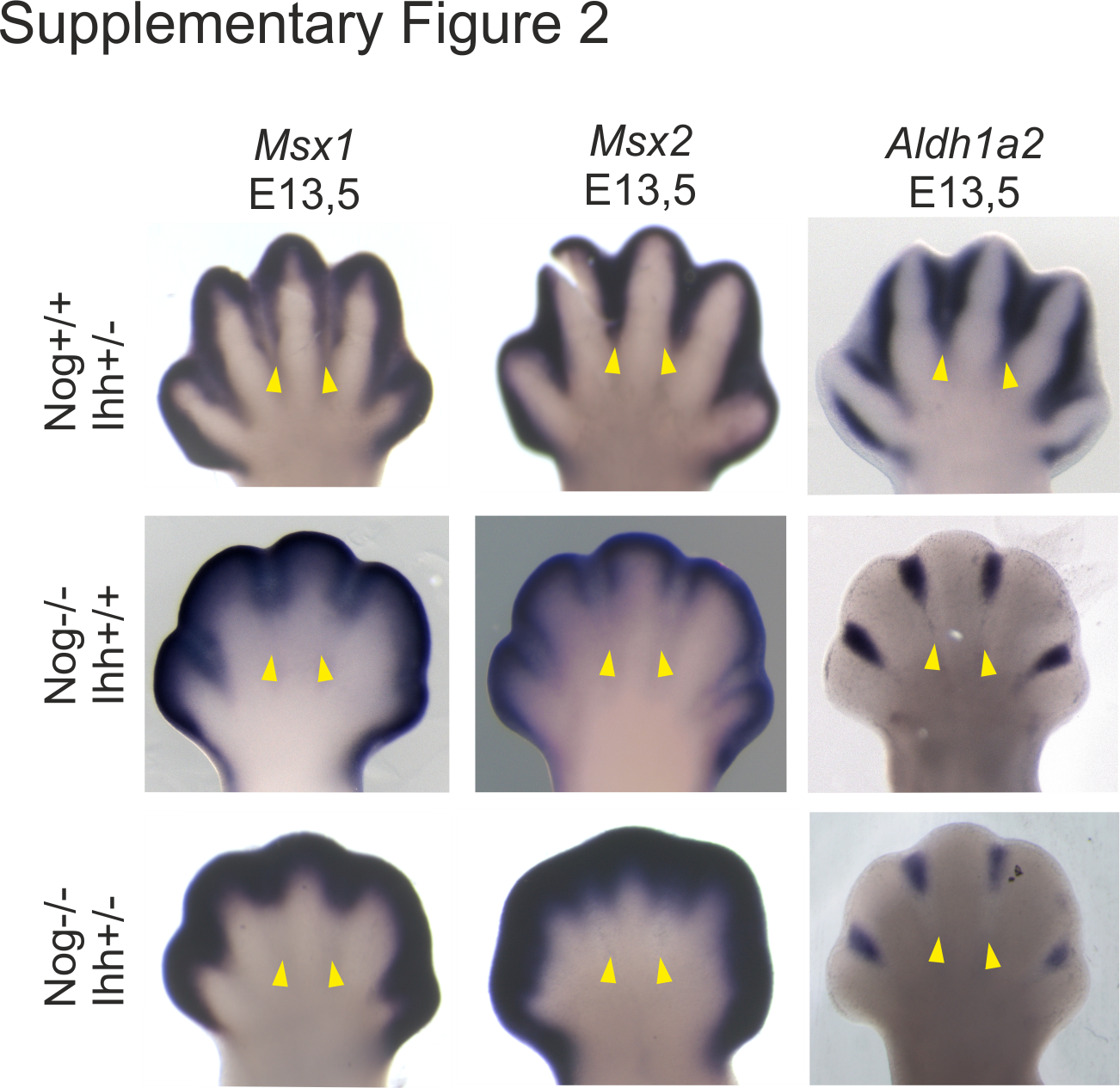

Supplement: S2 Fig — Nog+/+;Ihh+/- (control), Nog-/-;Ihh+/+ (normal Nog KO) and Nog-/-;Ihh+/- (compound mutant: Nog KO lacking one allele of Ihh) were analysed via whole-mount in-situ hybridisation for the expression of interdigit markers Msx1, Msx2 and Aldh1a2. No amelioration of the Nog KO phenotype can be seen in compound mutants. Proximal interdigital expression domains of Msx1, Msx2 and Aldh1a2 that are affected in Nog mutants are indicated by arrowheads. (TIF) [file pone.0197535.s002.tif]
